# Genomic insight into the nocturnal adaptation of the black-crowned night heron (*Nycticorax nycticorax*)

**DOI:** 10.1186/s12864-022-08904-y

**Published:** 2022-10-03

**Authors:** Haoran Luo, Site Luo, Wenzhen Fang, Qingxian Lin, Xiaolin Chen, Xiaoping Zhou

**Affiliations:** Key Laboratory of Ministry of Education for Coastal and Wetland Ecosystems, College of the Environment and Ecology, Xiamen University, Xiamen, 361102 People’s Republic of China

**Keywords:** Vision genes, Olfactory receptor genes, Nocturnal adaptation, Positive selection, Relaxed and intensified selection, Ardeidae birds

## Abstract

**Background:**

The black-crowned night heron (*Nycticorax nycticorax)* is an ardeid bird successfully adapted to the nocturnal environment. Previous studies had indicated that the eyes of the night herons have evolved several specialized morphological traits favoring nocturnal vision. However, the molecular mechanisms of the nocturnal vision adaptation of night herons remained inattentions. In this study, the whole genome of *N*. *nycticorax* was sequenced and comparative analyses were performed on the vision-related and olfactory receptor (OR) genes to understand the molecular mechanisms of the visual and olfactory adaptation of night herons.

**Results:**

The results indicated that a number of vision genes were under positive or relaxed selection in *N. nycticorax*, whereas a number of other vision genes were under relaxed or intensified selection in the boat-billed heron (*Cochlearius cochlearius*), which suggested that the two species adapt to nocturnality with different genetic mechanisms. The different selections acting on vision genes are probably associated with the enlargement of eye size and the enhancement of visual sensitivity in night herons. The analyses on olfactory receptor (OR) genes indicated that the total number of OR genes in the genomes of *N. nycticorax* and *C. cochlearius* were about half those in the little egret (*Egretta garzetta*), whereas the diversity of their OR genes was not remarkably different. Additionally, the number of expressed OR genes in the transcriptomes of *N. nycticorax* was also fewer than that in *E. garzetta*. These results suggest a reduced olfactory capability in night herons compared with *E. garzetta*.

**Conclusions:**

Our results provided evidence that several vision genes of the night herons were subjected to different natural selections, which can contribute to a better understanding of the genetic mechanisms of visual adaptions of the night heron. In addition, the finding of the reduced number of total and expressed OR genes in night herons may reflect a trade-off between olfaction and vision.

**Supplementary Information:**

The online version contains supplementary material available at 10.1186/s12864-022-08904-y.

## Introduction

Ardeid birds are foragers that rely on visual cues for the detection and capture of prey [1–3]. Most species within this family are diurnal, and few species, namely night herons, are nocturnal [1]. The black-crowned night heron (*Nycticorax nycticorax*) is the most common and widely distributed night heron that forages mostly at night but also forages in daytime, especially during the breeding season [4, 5]. Compared with diurnal ardeid species, the eyes of *N*. *nycticorax* have many anatomical characteristics similar to those of owls, such as relatively large size [6] and a tapetum lucidum behind the retina [7]. The rod-to-cone ratio of *N*. *nycticorax* has not yet been studied; however, many rod cells are found in the eyes of *N*. *nycticorax* [8], and high rod-to-cone ratio was reported in another night heron closely related to *N*. *nycticorax*, the yellow-crowned night heron (*Nyctanassa violacea*) [9]. Unlike owls, a large eye size does not give rise to larger binocular fields in *N*. *nycticorax* compared with diurnal ardeid species. The binocular field of *N*. *nycticorax* is vertically long and narrow with the bill, which was argued to be associated with their foraging technique rather than their nocturnal habits [6].

Recently, whole genomes have been sequenced for diverse nocturnal species from different animal groups, and the results have greatly advanced the understanding of the genetic mechanism underlying the sensorial adaptations of the nocturnal species. For instance, a comparative genomics analysis for the Chinese forest musk deer (*Moschus berezovskii*) indicated positively selected genes distributed in phototransduction and retinol metabolism pathways may be contributed to the nocturnality of this species [10]. Additionally, comparative genomic analyses of bats [11] and owls [12] revealed that adaptive evolution of the vision genes play important roles in the eye anatomical and physiological specializations favoring nocturnal vision. Other comparative genome analyses also provided evidence that some nocturnal species adapted to nocturnality by developing other sensorial modalities, such as olfaction and hearing. For example, the kiwi (*Apteryx mantelli*) genome has a remarkably high diversity of olfactory receptor genes, which proves that kiwis have well developed olfactory acuity [13, 14] and relies more on olfactory than other sensory systems for nocturnal foraging [15]. Furthermore, comparative analyses indicated that several evolutionary signatures associated with sensory adaptations to nocturnal environment are shared by at least two nocturnal bird groups of owls, chuck-will’s-widow (*Caprimulgus carolinensis*), and brown kiwi (*A. australis*) [16].

To date, only the genomes of two ardeid species, the little egret (*Egretta garzetta*) and the boat-billed heron (*Cochlearius cochlearius*), have been sequenced. *E*. *garzetta* is a diurnal species, and its genome shows an expansion of the olfactory receptor (OR) gene repertoire [17]. *C*. *cochlearius* is a strictly nocturnal forager; however, whether it relies on vision or tactile techniques for foraging is in conflict [18, 19]. The published genome has not been used to explore the genetic basis underlying the nocturnal adaptations in this species [20].

In the present study, the whole genome of *N*. *nycticorax* was sequenced, and comparative analyses of *E*. *garzetta*, *C*. *cochlearius*, and other birds within Pelecaniformes with high-quality genomes were performed. Previous phylogenetic studies indicated *N*. *nycticorax* is more closely related to *E*. *garzetta* than to *C*. *cochlearius*, suggesting nocturnal behavior of *N*. *nycticorax* and *C*. *cochlearius* have evolved independently [21]. We tested the natural selections acting on the vision genes of *N. nycticorax* and *C. cochlearius*, with the aim to improve the understanding of the molecular vision adaptations to nocturnality in night herons. The number of OR genes between *N*. *nycticorax* and *E*. *garzetta* at the genomic and transcriptome levels were also compared. The total number of OR genes in a genome are believed to be positively correlated to the olfactory bulb ratio of a species and therefore can be a good indicator for the olfactory abilities of the species [17]. The OR gene expansion in *E*. *garzetta* genome suggests enhanced olfactory abilities [17]. *N*. *nycticorax* has an olfactory bulb ratio similar to that of *E*. *garzetta* [22]. Here, we investigated whether the OR gene expansion is exhibited in *N*. *nycticorax*.

## Results

### Genome assembly and annotation

A total of 123.93 Gb (~ 97.4-fold coverage) high-quality sequences were obtained from five paired-end and mate-pair libraries (Table 1). The final size of the assembled *N*. *nycticorax* genome is 1179.04 Mb in length, which cover about 93% of the 1272.61 Mb genome size estimated by the 17 K-mer distribution (Supplement Figure S1). The contig and scaffold N50 sizes in our assembly are 58.55 and 3016.563 kb, respectively (Table 2), and the longest scaffold is 17,752 kb. The Core Eukaryotic Genes Mapping Approach (CEGMA) analysis indicated that 69.35% (172) complete and 12.5% (31) partial Core Eukaryotic Genes (CEGs) could be identified in our assembly. The Benchmarking Universal Single-Copy Orthologs (BUSCO) results showed that 93.0% complete (7724 single copies and 30 duplicated) and 2.2% (183) fragmented BUSCOs could be identified in our assembly, and 4.8% (401) were considered missing.

**Table 1:** Statistics of *N. nycticorax* genome sequencing

Pair-end libraries	Total data (Gb)	Read length (bp)	Sequence coverage (X)
230 bp	34.38	150	27.0
500 bp	35.85	125	28.2
2 k	24.23	125	19.0
5 k	14.78	125	11.6
10 k	14.70	125	11.5
Total	123.93		97.4

**Table 2:** Assembly statistics for *N. nycticorax* genome

Assembly	N50 (bp)	Number	Total Size (bp)
Contigs	58,550	46,539	1,116,931,249
Scaffolds	3,016,563	7,611	1,179,048,687

Contig numbers were contig after scaffolding. Details of the genome assembly statistics see Supplement Table S3

We observed that 8.39% of the whole assembly are repetitive sequences, which included 0.75% tandem repeat sequences, 0.66% DNA repeat elements, 5.15% long interspersed nuclear elements (LINE), 0.14% short interspersed nuclear elements (SINE), and 1.42% long terminal repeat elements (LTR). The functional annotation revealed that the assembly contains 13,361 predicted protein-coding genes. Among which, 13,358 (99.98%) predicted protein-coding genes were well annotated by the SwissProt, TrEMBL, Kyoto Encyclopedia of Genes and Genomes (KEGG), Gene Ontology (GO), and InterPro databases. Additionally, 335 candidate microRNA genes and 183 candidate tRNAs with total lengths of 28,473 and 13,666 bp, respectively, were also identified.

### Selective analysis of vision-related genes

**Fig. 1:**
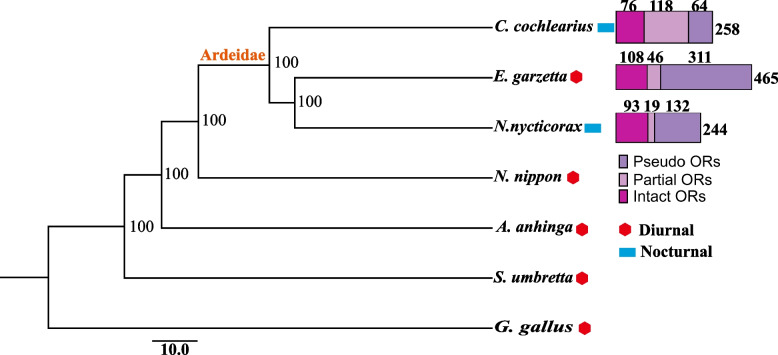
The maximum likelihood (ML) tree and the number of the OR genes in the genomes of the three ardeid species. The ML tree of *N*. *nycticorax* and other five Pelecaniformes birds based on 4121 single-copy orthologous genes. G. gallus was used as the outgroup species. The numbers above the bars represent the numbers of OR genes in *E*. *garzetta*, *C*. *cochlearius*, and *N*. *nycticorax* genome

To provide a phylogenetic frame of reference for selection analyses, we firstly constructed a maximum likelihood (ML) tree using the IQTREE based on the protein coding sequences of 4121 single-copy orthologous gene from *N*. *nycticorax*, *C*. *cochlearius*, *E*. *garzetta*, *Nipponia nippon*, *Anhinga anhinga*, *Scopus umbretta* and *Gallus gallus* (Fig. 1). Based on the ML tree, Phylogenetic Analysis by Maximum Likelihood (PAML) branch model, Branch-site Unrestricted Statistical Test for Episodic Diversification (BUSTED), and RELAX were used to detect signatures of selection in each of the 216 orthologous vision genes (Supplement Table S2.xlsx), which were yielded by EggNog and OrthoFinder searches and had sequences more than 99 bp in length.

From the 216 orthologous genes, a total of 21 positively selected genes were identified in *N*. *nycticorax*, of which three genes (*CCDC66*, *CDON* and *RPL24*) were identified by both PAML branch model and BUSTED, 18 genes were identified only by BUSTED (*CHD7*, *CRYBA1*, *CTNS*, *FAT3*, *IMPG1*, *IMPG2*, *MDM1*, *MFN2*, *MITF*, *OLFM3*, *OPN4*, *RRM1*, *SH3PXD2B*, *SKI*, *SLC7A11*, *UNC119*, *VEGFA*, and *WDR19*). In *C*. *cochlearius* lineage, no positively selected gene was identified by PAML branch model and one positively selected gene (*MED1*) was identified by BUSTED.

Because high rate of false positive in detection of positive selection due to relaxed selection, previous studies suggested using a combination of approaches to distinguish between positive and relaxed selection, arguing that a gene with both intensified and positive selection signatures likely constitutes targets of truly positive selection, while a gene with both relaxed and positive selection signatures likely has experienced relaxation of selective constraint [23, 24]. Among the positively selected genes mentioned above, 13 genes (*CCDC66*, *CDON*, *CHD7*, *CTNS*, *FAT3*, *IMPG2*, *MFN2*, *OPN4*, *RPL24*, *SH3PXD2B*, *SLC7A11*, *UNC119*, and *VEGFA*) in *N*. *nycticorax* were identified under intensified selection by the RELAX. Conversely, five genes (*CRYBA1*, *IMPG1*, *OLFM3*, *RRM1*, and *SKI*) in *N*. *nycticorax* and *MED1* in *C. cochlearius* were identified as under relaxed selection by both PAML branch model and RELAX.

In *N*. *nycticorax*, PAML branch model and RELAX both further identified *GNA11* under relaxed selection and *ATP8A2* under intensified selection, and respectively identified two (*GNAT1* and *PDGFRB*) and 15 genes (*ADAMTS18*, *EPAS1*, *FGFR2*, *FOXP2*, *GNAQ*, *HDAC1*, *HPS1*, *NHS*, *OPA1*, *PAX6*, *RPE65*, *SLC4A10*, *SMAD3*, *TTC8*, and *UCHL3*) under relaxed selection.

In *C. cochlearius*, RELAX further detected relaxed selection signatures in 13 additional genes (*ALDH1A3*, *CACNB2*, *CCDC66*, *EPAS1*, *FOXP2*, *JAG1*, *MAP3K1*, *OPA1*, *OPN4*, *PDE6B SLC4A10*, *SLC7A11*, *SMAD3*, and *SOX14*). Additionally, five (*CCDC66*, *CRB1*, *PDGFRB*, *RNF2* and *TULP3*) and seven genes (*ADAMTS18*, *CRB1*, *EPHB1*, *MEGF11*, *MYO7A*, *SLC25A25*, and *WDR19*) were respectively identified as under intensified selection by PAML branch model and RELAX, of which *CRB1* was shared by PAML branch model and RELAX.

Comparing between *N*. *nycticorax* and *C*. *cochlearius*, five genes (*EPAS1*, *FOXP2*, *OPA1*, *SMAD3*, *SLC4A10*) overlapped with similar selection signatures, while six genes (*ADAMTS18*, *CCDC66*, *OPN4*, *PDGFRB*, *SLC7A11*, and *WDR19*) overlapped with different or opposite selection signatures. The details of results of different selective analyses and gene functions are presented in Tables 3 and 4, and supplement Table 4.

**Table 3:** The results of selective analyses of the vision genes in *N*. *nycticorax*

Gene	PAML-ω0	PAML-ω1	PAML-P	PAML-Q	BUSTED-P	BUSTED-Q	RELAX-K	RELAX-P	RELAX-Q
*ADAMTS18*	0.2385	0.2385	5.158E-01	8.936E-01	0.5	0.46323	**0**	**0.005**	**0.0231**
*ATP8A2*	**0.2646**	**0.0001**	**9.826E-05**	**3.301E-03**	0.5	0.50859	**1.17**	**0.002**	**0.0136**
*CCDC66*	**0.3254**	**2.5296**	**1.639E-06**	**5.679E-05**	**1.054E-18**	**6.646E-17**	**3.86**	**6.22154E-14**	**1.730E-12**
*CDON*	**0.2713**	**1.0674**	**0**	**0**	**4.277E-18**	**1.796E-16**	**10.54**	**2.398E-26**	**1.335E-24**
*CHD7*	0.0817	0.0817	9.238E-01	9.900E-01	**1.837E-20**	**2.315E-18**	**35.59**	**0.0001**	**0.0015**
*CRYBA1*	**0.0477**	**0.2343**	**2.176E-04**	**4.310E-03**	**0.0001**	**0.0013**	**0.32**	**3.122E-06**	**3E-05**
*CTNS*	0.4024	0.4024	6.838E-02	3.833E-01	**0.0011**	**0.0108**	**10.39**	**0**	**0**
*EPAS1*	0.2276	0.2276	8.291E-01	1	0.5	0.4295	**0.11**	**0.002**	**0.008**
*FAT3*	0.0913	0.0913	8.275E-02	3.955E-01	**1.030E-09**	**2.165E-08**	**50**	**1.99E-11**	**2.768E-10**
*FGFR2*	0.0738	0.0738	3.157E-01	6.267E-01	0.5	0.4632	**0.44**	**0.003**	**0.0151**
*FOXP2*	0.1829	0.1829	1.735E-01	4.966E-01	0.5	0.4632	**0**	**0.011**	**0.0394**
*GNA11*	**0.0001**	**0.3321**	**6.565E-04**	**1.137E-02**	0.5	0.4632	**0.15**	**0.008**	**0.0318**
*GNAQ*	0.1753	0.1753	4.591E-02	2.204E-01	0.5	0.5085	**0**	**0.004**	**0.0218**
*GNAT1*	**0.0217**	**0.1128**	**5.745E-04**	**1.025E-02**	0.5	0.525	0.89	0.648	1
*HDAC1*	0.0411	0.0411	1.530E-01	4.903E-01	0.5	0.4632	**0**	**0.017**	**0.05**
*HPS1*	0.1729	0.1729	9.458E-01	9.900E-01	0.019	0.1408	**0.61**	**0.01**	**0.0371**
*IMPG1*	**0.2561**	**0.6748**	**2.942E-04**	**4.943E-03**	**8.481E-12**	**2.760E-10**	**0.5**	**8.008E-13**	**2.186E-11**
*IMPG2*	0.3791	0.3791	7.494E-01	1	**2.423E-11**	**2.290E-10**	**39.63**	**0.013**	**0.0464**
*MDM1*	0.3542	0.3542	9.218E-01	9.900E-01	**0.002**	**0.018**	1.18	0.476	0.6307
*MFN2*	0.0505	0.0505	2.719E-01	5.703E-01	**8.329E-05**	**0.0011**	**5.49**	**6.713E-05**	**0.0006**
*MITF*	0.0183	0.0183	7.086E-01	9.484E-01	**4.630E-10**	**1.167E-08**	7.68	0.761	0.8066
*NHS*	0.1380	0.1380	1.199E-01	4.903E-01	0.5	0.4632	**0.09**	**0.001**	**0.0055**
*OLFM3*	**0.1720**	**0.9096**	**4.110E-07**	**9E-06**	**3.985E-10**	**2.510E-09**	**0**	**1.894E-12**	**3.373E-11**
*OPA1*	0.1855	0.1855	6.913E-02	3.833E-01	0.031	0.2055	**0**	**0.001**	**0.0055**
*OPN4*	0.0361	0.0361	5.956E-02	3.589E-01	**0.0001**	**0.0014**	**13.37**	**9.919E-13**	**2.208E-11**
*PAX6*	0.1595	0.1595	9.914E-01	9.900E-01	0.5	0.4632	**0.15**	**0.007**	**0.0288**
*PDGFRB*	**0.0564**	**0.4014**	**1.792E-04**	**4.139E-03**	0.5	0.4632	5.54	0.853	0.8183
*RPE65*	0.1523	0.1523	1.923E-01	5.030E-01	0.5	0.4632	**0.09**	**0.001**	**0.0055**
*RPL24*	**0.0089**	**1.4164**	**0**	**0**	**1.963E-07**	**3E-06**	**29.42**	**6.06E-12**	**9.635E-11**
*RRM1*	**0.0198**	**0.6076**	**8.728E-06**	**2.419E-04**	**0.0024**	**0.0209**	**0.07**	**0.002**	**0.0106**
*SH3PXD2B*	0.1368	0.1368	5.E-02	3.300E-01	**1.790E-10**	**5.641E-09**	**17.08**	**1.529E-12**	**2.838E-11**
*SKI*	**0.0631**	**0.6871**	**0**	**0**	**8.824E-08**	**1.5E-06**	**0.2**	**3.898E-23**	**1.446E-21**
*SLC4A10*	0.1424	0.1424	3.260E-01	6.365E-01	0.417	0.4632	**0**	**9.367E-05**	**0.0008**
*SLC7A11*	0.2571	0.2571	9.602E-01	1	**7.536E-05**	**0.0003**	**2.37**	**0.001**	**0.0059**
*SMAD3*	0.0281	0.0281	7.100E-01	9.484E-01	0.5	0.4632	**0**	**0.0002**	**0.0021**
*TTC8*	0.2426	0.2426	2.313E-02	1.554E-01	0.5	0.5085	**0**	**0.001**	**0.0091**
*UCHL3*	0.1590	0.1590	1.460E-01	3.774E-01	0.5	0.5085	**0**	**0.007**	**0.0318**
*UNC119*	0.0264	0.0264	4.437E-03	6.833E-02	**0.001**	**0.0105**	**50**	**0.006**	**0.0256**
*VEGFA*	0.6017	0.6017	8.976E-01	9.9E-01	**0.003**	**0.0236**	**46.91**	**3.583E-05**	**0.0003**
*WDR19*	0.1118	0.1118	4.362E-01	1	**5.819E-21**	**1.099E-19**	0.97	0.889	0.85

Significant results were highlighted in bold

**Table 4:** The results of the selective analyses of the vision genes in *C. cochlearius*

Gene	PAML-ω0	PAML-ω1	PAML-P	PAML-Q	BUSTED-P	BUSTED-Q	RELAX-K	RELAX-P	RELAX-Q
*ADAMTS18*	0.2385	0.2385	4.639E-03	8.160E-02	0.5	5.21E-01	**1.16**	**0.002**	**0.0184**
*ALDH1A3*	0.0315	0.0315	8.359E-02	4.524E-01	0.002	8.39E-02	**0.32**	**0.006**	**0.0378**
*CACNB2*	0.1851	0.1851	1.489E-01	5.051E-01	0.5	5.21E-01	**0**	**0.001**	**0.0108**
*CCDC66*	**0.5134**	**0.0175**	**2.815E-06**	**9.902E-05**	0.5	5.21E-01	1	1	0.8932
*CRB1*	**0.1249**	**0.0212**	**3.556E-07**	**1.668E-05**	0.0035	8.39E-02	**2.51**	**5.349E-05**	**0.0011**
*EPAS1*	0.2276	0.2276	1.937E-01	6.102E-01	0.5	5.00E-01	**0**	**0.001**	**0.0073**
*EPHB1*	0.0485	0.0485	2.102E-02	2.201E-01	0.5	5.21E-01	**15.9**	**1.384E-05**	**0.0003**
*FOXP2*	0.1829	0.1829	9.362E-02	4.841E-01	0.5	5.21E-01	**0**	**0.005**	**0.0374**
*JAG1*	0.0328	0.0328	1.439E-01	5.051E-01	0.5	5.21E-01	**0**	**0.007**	**0.0418**
*MAP3K1*	0.2610	0.2610	5.521E-02	3.237E-01	0.5	5.21E-01	**0**	**0.001**	**0.0108**
*MED1*	**0.0319**	**0.1132**	**1.036E-03**	**2.429E-02**	6.801E-05	**9.64E-03**	**0.64**	**0.0004**	**0.0062**
*MEGF11*	0.1747	0.1747	2.070E-02	2.201E-01	0.5	5.21E-01	**46.41**	**0.002**	**0.0184**
*MYO7A*	0.0001	0.0001	1	1	0.5	5.00E-01	**15.7**	**0.006**	**0.0176**
*OPA1*	0.1855	0.1855	3.263E-02	3.061E-01	0.5	5.21E-01	**0**	**7.505E-06**	**0.0002**
*OPN4*	0.0361	0.0361	1.507E-01	5.051E-01	0.5	5.21E-01	**0**	**5.853E-07**	**3.503E-05**
*PDGFRB*	**0.0754**	**0.0077**	**4.477E-04**	**1.260E-02**	0.5	5.21E-01	1.12	0.594	0.7279
*RNF2*	**0.0442**	**0.0203**	**0**	**0**	0.5	5.21E-01	1.21	0.88	0.8917
*SLC25A25*	0.0099	0.0099	4.839E-01	7.612E-01	0.003	8.39E-02	**18.88**	**7.371E-10**	**8.822E-08**
*SLC4A10*	0.1424	0.1424	3.054E-01	6.319E-01	0.5	5.21E-01	**0**	**8.979E-05**	**0.0015**
*SLC7A11*	0.2571	0.2571	4.125E-03	9.096E-02	0.5	5.00E-01	**0**	**0.002**	**0.0098**
*SMAD3*	0.0281	0.0281	5.467E-01	8.013E-01	0.5	5.21E-01	**0**	**0.0003**	**0.0054**
*SOX14*	0.0151	0.0151	9.751E-01	1	0.5	5.21E-01	**0**	**7.505E-06**	**0.0002**
*TULP3*	**0.2277**	**0.1016**	**7.272E-230**	**5.116E-228**	0.02	4.05E-01	1.58	0.131	0.3038
*WDR19*	0.1118	0.1118	4.995E-02	3.671E-01	0.5	5.00E-01	**2.3**	**0.003**	**0.0110**

Significant results were highlighted in bold

### Olfactory receptor genes analysis

The total number of OR genes identified from *N*. *nycticorax*, *C*. *cochlearius*, and *E*. *garzetta* were 244, 258, and 465, respectively. The proportions of intact, partial, and pseudo OR genes were 38.1% (93), 7.8% (19), and 54.1% (132) in *N*. *nycticorax*; 29.5% (76), 45.7% (118), and 24.8% (64) in *C*. *cochlearius*; and 23.2% (108), 9.9% (46), and 66.9% (311) in *E. garzetta* (Fig. 1). *E*. *garzetta* had the highest number of total and intact OR genes but the lowest proportion of intact OR genes among the three species owing to the large number of pseudogenes.

The intact OR genes in *N*. *nycticorax*, *C*. *cochlearius* and *E. garzetta* could be classified into 13, 7, and 10 subfamilies, respectively. As previously reported for other birds [17], the OR14 subfamily were the most abundant in all the three species, *n* = 61, *n* = 65, and *n* = 75, respectively (Table 5).

**Table 5:** Classes of Intact ORs

Classes	Subfamily	*N. nycticorax*	*C. cochlearius*	*E. garzetta*
Type I-class II (γ)	1			
	2	3	1	3
	4	6	1	6
	5	5	5	6
	6	4	1	3
	7			
	8	1		
	9			
	10	1	1	3
	11	2		2
	13			
	14	61	65	75
	total	83	74	98
Type I-class I	51	2		1
	52		2	7
	55	4		
	56			
	total α			8
	δ	6		
	ε	1		
	ζ			
Type II	η	1		2
	θ	2		

The average Shannon entropy (*H*) values estimated from the Type I Class II (γ) OR genes were 0.492 ± 0.414 in *N*. *nycticorax*, 0.499 ± 0.413 in *C*. *cochlearius*, and 0.489 ± 0.430 in *E. garzetta*. The Wilcoxon signed-rank test indicated that the two night herons had no significantly higher *H* values than *E. garzetta* (*P* = 0.490 in *N*. *nycticorax* vs. *E. garzetta*, *P* = 0.376 in *C*. *cochlearius* vs. *N*. *nycticorax*).

To test positive selection in the OR14 subfamily, Genetic Algorithm Recombination Detection (GARD) was firstly used to detect the presence of recombination in the OR14 genes. The results revealed two breakpoints at the alignment nucleotide positions 509 and 719 in *E. garzetta*; five breakpoints at positions 207, 331, 464, 573, and 642 in *N*. *nycticorax*; and three breakpoints at positions 369, 507, and 631 in *C*. *cochlearius*. Based on the inferred recombination breakpoints, Single-likelihood Ancestor Counting (SLAC), Mixed Effects Model of Evolution (MEME), and Fast Unconstrained Bayesian Approximation (FUBAR) were used to infer signatures of positive selection and all three analytical methods identified positive selection in amino acid positions 52 (near transmembrane domain (TM) 2), 107 (in TM3), 196 (closest site near TM5), 204 (in TM5), 251 (in TM6), and 283 (in TM7) in *E. garzetta* (Supplement Table S5). Additionally, positive selection was identified in amino acid positions 93 (the closest site near TM3) and 110 (in TM3) in *N*. *nycticorax* (Supplement Table S6) and in amino acid positions 16, 47, 107 (in TM3), 172, 196 (closest site near TM5), 204 (in TM5), 207 (in TM5), and 283 (in TM7) in *C*. *cochlearius* (Supplement Table S7) by all three analytical methods. These results indicated that *E. garzetta* and *C*. *cochlearius* share four positive selection positions, and the other positions were species specific.

**Fig. 2:**
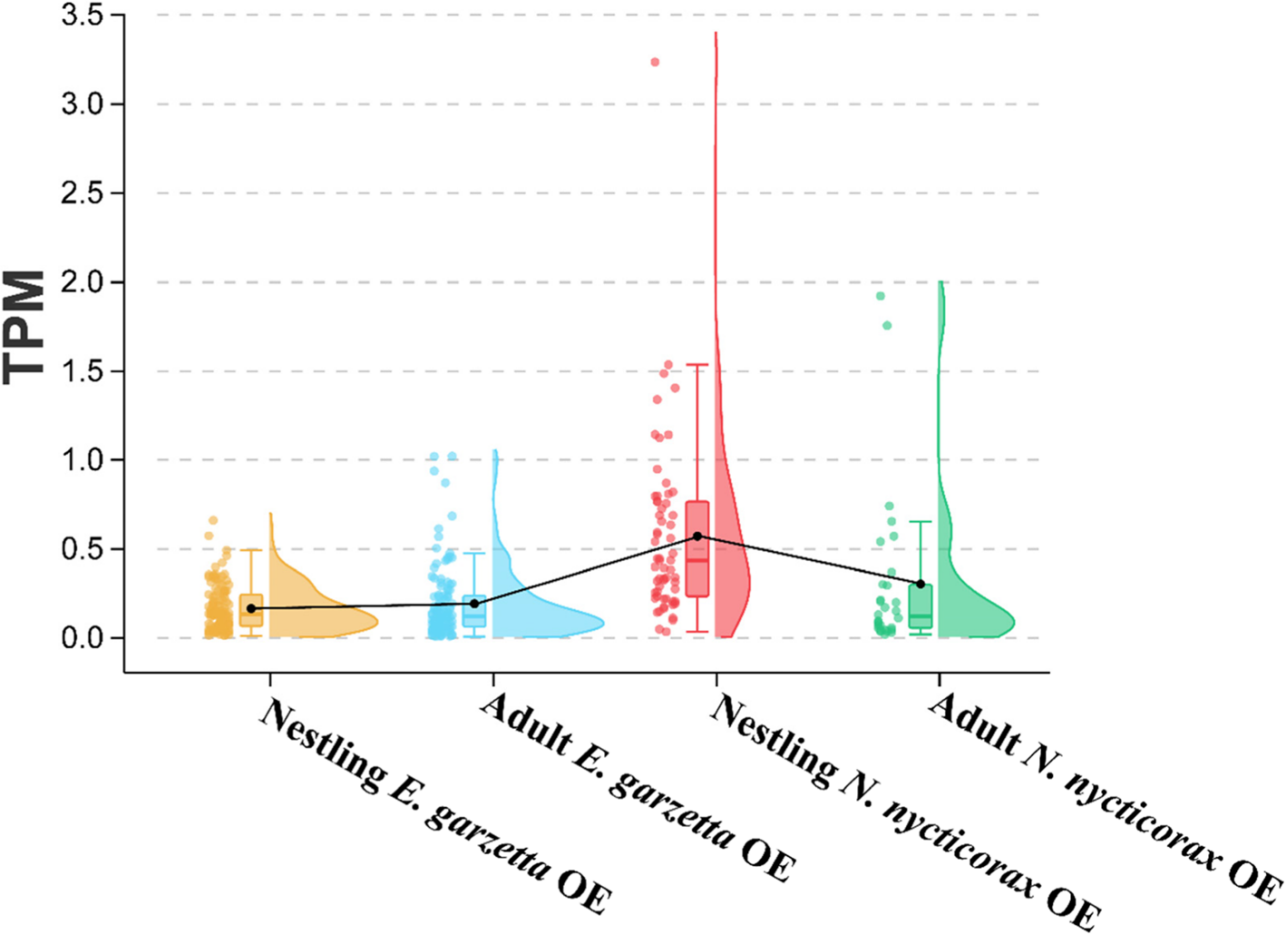
Raincloud plot of ORs expression levels. Each plot represents the expression levels of ORs. Quartiles are shown in the error bars, and the cloud showed the expression distributions. Black dots in the error bars represent the mean values of each group, black line was used to connection mean values

The transcriptomes of the olfactory epitheliums (OEs) of *N*. *nycticorax* and *E. garzetta* were studied to confirm that the identified intact OR genes were actually expressed. The results indicated that 61 genes (61 in nestlings and 30 in adults) had detectable expression in *N*. *nycticorax*, and 132 OR genes (113 in nestlings and 118 in adults) had detectable expression in *E. garzetta*. Notably, the nestling *N*. *nycticorax* expressed more OR genes than the adults, where 31 of the 61 expressed genes had no detectable expression in adult OEs. In *E. garzetta*, 14 expressed OR genes had no detectable expression in nestling OEs, and 19 had no detectable expression in adult OEs. The expression levels of ORs were quite low in both species, wherein the average expression levels ranged from 0.161 transcripts per kilobase million (TPM) in the nestling *E. garzetta* OE to 0.566 TPM in the nestling *N*. *nycticorax* OE (Fig. 2, Supplement Fig. 2, and Supplement Table S3.xlsx).

## Discussion

This study presents the first genome assembly of *N*. *nycticorax* and attempted to uncover the potential molecular basis that underlies the adaptation of night herons’ visual and olfactory systems to nocturnality. Consistent with the expectation that night herons would show signatures of adaptive evolution associated with their visual systems, we detected different selective pressures acting on a number of different vision genes in *N*. *nycticorax* and *C*. *cochlearius.* Additionally, we found relatively higher number of genes with positive selection and relaxed selection in *N*. *nycticorax*, whereas relatively higher number of genes with intensified selection in *C*. *cochlearius*. These results indicated different evolutional mechanisms of visual adaptation between *N. nycticorax* and *C. cochlearius*, which were possibly due to that the nocturnalities of *N*. *nycticorax* and *C*. *cochlearius* are of independent origin, and *N*. *nycticorax* and *C*. *cochlearius* have different diel activity patterns and degrees of dependence on vision [4, 19, 21].

Adaptive evolution of the vision genes facilitated by positive selection was widely reported in many other animals which rely on vision in dim-light environments, such as mole rats [25], Chinese forest musk deer [10], okapi [26], bats [11], and owls [27], although the positively selected genes varied cross species. Similar to these findings, 16 vision genes were found to undergo positive selection in *N*. *nycticorax*, suggesting that positive selection has played an important role in the adaptive evolution of the vision genes and thereby contributed to the morphological or functional modifications of the eyes favoring night vision. Specifically, *CDON* and *MITF* are associated with eye size, deleterious mutations of which have been correlated with microphthalmia [28, 29]. *CTNS*, *MFN2*, and *SH3PXD2B* are involved in the development and maintenance of cornea and lens [30–34]. Positive selections in these genes may be responsible for the enlarged eye size, cornea, and lens of *N*. *nycticorax*, which allow more light to enter the eyes in dim conditions [35, 36]. The remaining positively selected genes are all retina-associated, involved in the development and maintenance of retina (*FAT3*, *OPN4*, *SLC7A11*, *VEGFA*) [37–41] and photoreceptor (*CHD7*, *CCDC66*, *IPMG2*, *WDR19*) [42–45]. Positive selections in these genes likely play important roles in the retinal modifications, such as the preponderance of rods over cones [9].

In addition to positive selection, 23 genes in *N*. *nycticorax* and 14 genes in *C*. *cochlearius* were identified as under relaxed selection, suggesting that relaxed selection is the predominant force that shaped the evolution of visual adaptation in both species. For example, *GNAT1* in *N*. *nycticorax* and *PDE6B* in *C*. *cochlearius* are involved in the rod phototransduction pathway [46, 47], which is responsible for dim light vision [27, 48]. Relaxed selection of these two genes may have allowed them to explore new functions increasing visual sensitivity in *N*. *nycticorax* and *C. cochlearius,* respectively. The other relaxed selection genes, such as the five genes shared by *N*. *nycticorax* and *C*. *cochlearius*, are associated with morphologies or functions of (*FOXP2*) [49], lens (*SMAD3*) [50], cornea (*SLC4A10*) [51], retina (*EPAS1*) [52], and optic nerve (*OPA1*) [53, 54], respectively. Relaxed selection of these genes may contribute to adjust the morphologies or functions of different eye components to enhance night vision as the finding in a previous study of barn owl (*Tyto alba*) [35]. In the barn owl, the unique phenotypes of the scotopic-adapted eyes are hypothesized to have evolved through relaxed or intensified selection in genes with predominant roles in different eye elements [35]. Additionally, the authors suggested that some genes have coevolved with contrasting selective signature due to adaptive compensation [35]. *N*. *nycticorax* and *C*. *cochlearius* separately have one and 11 genes with signatures of intensified selection, which may also be explained by the same evolutionary mechanism.

Turning to the olfactory receptor gene repertoires, the total number and genetic diversity of ORgenes are commonly used as proxy for olfactory capability [55–57]. Fewer number of OR genes in the genomes of *N*. *nycticorax* and *C*. *cochlearius* along with fewer number of expressed OR genes in the transcriptomes of *N*. *nycticorax* reflected reduced olfactory capabilities in night herons compared with *E*. *garzetta*. Night heron reduced olfactory capabilities may be a trade-off with their large eyes [58–60], because large eyes are energetically costly to develop and maintain [61]. An animal with increased investment in vision modality could decrease investment in other sensory modalities, including olfaction [62].

The transcriptome results showed that the number of expressed OR genes in *E*. *garzetta* is higher than the number of intact genes annotated in the genome. This finding reflected that some functional genes were identified as partial OR genes because of the genome assembly quality or the expression of some pseudogenes. The transcription of OR pseudogenes in the OE was also reported in humans [63] and other animals [64, 65]. Conversely, the number of expressed genes in *N*. *nycticorax* is fewer than the number of intact genes annotated in the genome, revealing that a part of the OR gene is transcriptionally inactive [66]. Additionally, some OR genes in *N*. *nycticorax* and *E*. *garzetta* are specifically expressed in nestling or adult samples, which suggests that these OR genes play different roles in specific developmental stages. Similar findings were also reported in other animals, such as the Chinese perch (*Siniperca chuatsi*) [65] and the Leach's storm-petrel (*Oceanodroma leucorhoa*) [67].

The expression of OR genes in the OE of *N. nycticorax* and *E. garzetta* indicated that they play olfactory roles despite of their low expression levels. The study of Leach's storm-petrel [67], a highly olfactory forager, also indicated the low expression levels of OR genes in the OE. Therefore, the low expression levels of OR genes may be common in birds. Additionally, the positively selective sites exhibited in the expanded OR14 subfamily in the night herons and *E. garzetta* further supported that OR genes are ecologically functional, consistent with the findings in other birds [16].

## Conclusions

This study provides the first analysis of the nocturnal adaptation of night herons from vision-related and OR genes. The different selective signatures detected in the vision genes provided evidence that the visual systems of the night herons have undergone adaptive evolution for enhanced night vision. Additionally, adaptive evolution of the vision genes in *N*. *nycticorax* is predominantly driven by positive and relaxed selections, while that in *C*. *cochlearius* is predominantly driven by relaxed and intensified selections. The comparative analyses of OR gene repertoires in the genomes and transcriptome between night herons and *E. garzetta* indicated reduced olfactory capacity in night herons, but olfactory capacity of night herons still has ecological adaptation. Future studies on olfactory function in different life activities in night herons and other diurnal egrets would provide a better understanding of the roles of OR genes in ardeid birds.

## Materials and methods

### Sample collection

*N*. *nycticorax* and *E. garzetta* were captured in Xiamen, Fujian, China. Each species includes three nestlings and three adults. To reduce potential kinships between samples, each nestling was sampled from different nest and adult samples were collected from a location different from the nestlings. The body lengths of the nestlings of each species were less than 15 cm. The adults were identified by their breeding plumage. Muscle sample was collected from one *N*. *nycticorax* and stored at − 80 °C until DNA extraction for whole-genome sequencing. Olfactory epithelium (OE) tissues were collected from each individual and stored in RNAlater (Invitrogen, Vilnius, Lithuania) at 4 °C until RNA extraction.

### DNA and RNA extraction and sequencing

Genomic DNA was extracted using QIAGEN® Puregene Tissue Core Kit A according to the manufacturer's instructions (Qiagen, Beijing, China). Two short insert libraries (230 and 500 bp) were constructed using the Illumina TruSeq DNA Library Preparation Kit (Illumina, San Diego, USA), and three mate pair libraries (2, 5, and 10 kbp) were constructed using the Nextera Mate Pair Sample Preparation Kit (Illumina, San Diego, USA). The libraries were sequenced on the Illumina HiSeq 2500 sequencing platform at Novogene (Beijing). Cutadapt [68] was used to remove the adaptor in the sequencing data and Trimmomatic [69] was used to remove low-quality reads with a minimum quality score of 20.

RNA was extracted from each OE sample using Invitrogen TRIzol (Invitrogen, Vilnius, Lithuania). The 150 bp paired-end RNA sequencing libraries were generated using the NEBNext Ultra RNA Library Prep Kit (New England Biolabs, Ipswich, MA, USA). All libraries were sequenced on the Illumina HiSeq 2500 sequencing platform at Novogene (Beijing). FastQC was used to evaluate the sequencing quality, and Trimmomatic [69] was used to remove adaptors and low-quality reads. The clean data for each sample was over 6 GB.

### Genome assembly

Jellyfish [70] was used to count of k-mers from cleaned sequencing reads to estimate genome size of *N*. *nycticorax* genome. The shotgun assembly of *N*. *nycticorax*’s whole genome was performed using SOAPdenovo2 [71]. All assembly steps were performed by following the official guideline, including read error correction, de Bruijn graph construction using short-insert-size library data, contig construction, realignment of the linkages between the contigs, and the creation of scaffolds from the short-insert-size paired ends and long-distance paired ends. The completeness of the assembled genome was assayed using Core Eukaryotic Genes Mapping Approach (CEGMA) [72], using 248 highly conserved core eukaryotic genes (CEGs). The completeness of the assembled genome was also evaluated by Benchmarking Universal Single-Copy Orthologs (BUSCO) v5.1.3 [73] with Hmmsearch (hmmer.org) 3.1 and Aves (odb10) dataset, which contains 8338 universal single-copy genes in birds.

### Genome annotation

Repetitive sequences in *N*. *nycticorax* genome were identified by de novo and homology-based approaches. In the de novo approach, RepeatModeler (https://www.repeatmasker.org) was used to identify interspersed repeats and generate de novo repeat libraries. RepeatMasker (https://www.repeatmasker.org) was run with the de novo libraries to identify interspersed repeats. Tandem Repeats Finder [74] was used to identify tandem repeats. In the homolog-based approach, RepeatMasker was used to search the interspersed repeats against the Repbase2 repeat database. Repeated proteins were identified by using RepeatProteinMasker.

To predict protein coding genes, proteins of *Homo sapiens*, *Taeniopygia guttata*, *G. gallus*, and *Meleagris gallopavo* were downloaded and aligned to *N*. *nycticorax* genome using TBLASTN [75] with an E-value cutoff of 1E-5. Subsequently, the homologous sequences of *N*. *nycticorax* genome were aligned against the matching proteins using Genewise2 [76] to predict the gene structure. Functions of the protein coding genes were annotated by blasting in KEGG [77], GO [78], SwissProt [79], and TrEMBL [79] databases. Gene domains were determined by InterProScan [80]. Moreover, tRNAscan-SE [81, 82] and INFERNAL [83] were used to annotate microRNAs and tRNAs in *N*. *nycticorax* genome.

### Selection of vision-related genes

The protein sequences of other Pelecaniformes birds (*E. garzetta* [GCA_000687185.1], *N. nippon* [GCA_000708225.1], *C. cochlearius* [VWPP00000000], *A. anhinga* [WBMU00000000], and *S. umbretta* [VZTL00000000]) and *G. gallus* (GRCg6a-GCA_000002315.5) were downloaded from Birds 10 K Phase I and II databases on the basis of sequence depth higher than 50 × and the availability of official predicted protein sequences. OrthoFinder2 [84] was used to search single-copy orthologous genes in *N*. *nycticorax* and the six birds with an E-value of 1e − 10 in the all-against-all BLASTP step. A total of 4121 single-copy orthologous genes were obtained and used to construct a maximum likelihood (ML) tree with IQTREE [85] with substitution model automatically selected by ModelFinder [86]. The ML tree (Fig. 1) was used in the downstream nature selection analysis of vision-related genes.

The orthologous genes of the seven birds involved in “eye development processes” (GO:0001654), “visual perception” (GO:0007601), “detection of light stimulus” (GO:0009583), and the rod phototransduction pathway (KEGG map04744) were re-annotated by EggNog-mapper V2.1.2 [87] with hmmer [88] search engine (E-value 1e-5) in EggNog V5 Aves (8782) database [89] and Basic Local Alignment Search Tool (BLAST) search engine [90] in the NR database. The results obtained 299 vision-related orthologous groups from the EggNOG and NR databases (Supplement Table S1.xlsx). The nucleotide sequences of the orthologous genes were aligned using ParaAT 2.0 [91] and alignments less than 99 bp (33AA) were filtered out. Finally, there was 216 candidate vision related orthologous genes remaining for the test of selections, of which, 159 orthologous genes had sequences for all the seven birds and 57 genes missed in one species of *N. nippon*, *C. cochlearius*, *A. anhinga*, or *S. umbretta*. For each orthologous gene, if multiple transcript variants were available in a species, the longest CDS of these orthologous genes were used in the downstream analysis of the selection.

Based on the ML tree, the branch model in the CodeML in PAML [92] was used to test for signatures of the positive selection acting on the vision genes of *N*. *nycticorax* and *C*. *cochlearius*, which were separately labeled as foreground branch using EasyCodeML [93]. The branch model tests one ratio model (same ω ratio of all branches) against two ratio model (different ω ratios between foreground background branches) based on likelihood ratio tests (LRT). The P-values calculated by LRT were then adjusted by false discovery rate (FDR) correction [94] with a cutoff of 0.05. Genes with ω1 higher than 1 and FDR < 0.05 were considered as positive selection genes.

Considering that positive selection sometimes acts only on a few sites and within a short evolutionary time period, we also used BUSTED [95] in Hyphy [96] to identify the vision related gene with evidence of positive selection at a fraction of sites. For analyses, BUSTED classifies sites to three rate classes (ω1 ≤ ω2 ≤ 1 ≤ ω3) representing strong and weak conservation and positive selection and estimates the proportion of sites belonging to each ω class occurring in both foreground and background branches (unconstrained model). Positive selection is then detected by comparing unconstrained model to constrained model (null model, ω3 = 1 on the foreground branch), and the significance was tested using LRT. For our analyses, *N*. *nycticorax* and *C*. *cochlearius* branch were respectively used as the foreground branch and the remaining branches were treated as the background branches. FDR correction was used to adjust *P*-values. Genes with adjusted *P*-values less than 0.05 and ω3 in foreground branch significantly greater than background branches were assigned as positive selected genes.

We further used two methods were used to test relaxed or intensified selection acting on the night herons. The first method was followed the procedure described in the barn owl research [35]. Briefly, a significantly higher ωForeground than ωBackground (*P* < 0.05, FDR < 0.05) in the branch model test suggests a relaxed selection in the foreground branch. A ωForeground significantly lower than ωBackground suggests the intensified selection in the foreground branch. The second method was RELAX [24] in HyPhy [96], based on the branch-site model. RELAX tests for relaxed or intensified selection were performed using parameter *k*, where *k* > 1 suggests intensified selection in the test branch and that the distribution of ω categories is close to 1 compared with that of the background, whereas *k* < 1 suggests relaxed selection in the test branch and that the distribution of ω categories is far from 1. The setting of test branch and background branch was the same in all the approaches,

### OR genes annotation and analysis

A custom BLAST database was created using the known nucleotide sequences of OR genes downloaded from *H. sapiens*, *Canis lupus familiaris*, *Bos taurus*, *T. guttata*, *G. gallus*, *Anolis carolinensis*, and *M. gallopavo* to identify the OR genes in genomes of *N*. *nycticorax*, *C*. *cochlearius*, and *E. garzetta*. The genomes of the three ardeid birds were aligned to the custom database using TBLASTN [75] with an E-value cut-off of 10. A candidate OR gene that best hit with the smallest E-value was retained. Genewise2 [76] was used to search 750 bp upstream and 750 bp downstream of the retained candidate ORs for finding the open reading frame (ORF). Based on the Genewise results, the candidate OR genes with normal start codons, stop codons, and more than 650 bp size that can code for seven transmembrane (TM) domains were identified as intact genes, the candidate OR genes without a start and/or stop codon were identified as partial genes, and the candidate OR genes with frameshift mutations and or premature stop codons were identified as pseudogenes. Subsequently, OR genes were distinguished from non-OR G protein-coupled receptors (GPCRs) using the neighbor-joining tree constructed in MEGA X software [97] using the candidate intact OR sequences and six non-OR GPCR sequences from *Frizzled* [98]. The candidate intact ORs that were clustered with non-OR GPCRs were filtered out. The candidate partial and pseudo-OR genes were subjected to BLAST, and those that had the top BLAST hits to non-OR sequences were omitted.

The Shannon entropy (*H*) [99] of Type I Class II (γ) OR genes was calculated by BioEdit [100] to investigate the diversity of ORs in the three ardeid birds. The Type I Class II (γ) OR sequences of the three ardeid birds were aligned using the Muscle program [101]. Gaps were excluded, and *H* was separately averaged across all positions for each ardeid species. *H* ≥ 2.0 is considered a variable position, *H* ≤ 2 is considered a conserved position, and *H* ≤ 1.0 is considered a highly conserved position [102].

Because phylogeny-based selection detection methods tend to overestimate the extent of positive selection among members of multigene families due to the presence of recombination or gene conversion [103], the analysis of positively selective sites on genes in OR family 14 were performed on the Datamonkey website [104] using the methods that allow to test for positive selection in the presence of recombination. Specifically, GARD [105] was used to generate multiple phylogenies based on putative nonrecombinant fragments to avoid the misleading estimation of selection caused by recombination and gene conversion. Then, SLAC [106], MEME [107], and FUBAR [108] methods were used in HyPhy (2.5.32) [95] to infer the signatures of the positive selection. Sites were considered under positive selection when they were detected by all the three methods with *Q* < 0.05 in MEME, *P* < 0.1 in SLAC, and Bayesian posterior probability > 0.95 in FUBAR. TM–helix in OR14 was predicted by TOPCONS [109]. Site positions followed *G. gallus* XM_001236559.6 (NCBI *G. gallus* annotation release 105-GRCg7b, chromosome assembly level) by sequence alignments.

### OR gene expression analysis

HISAT2 [110] was used to map the high-quality reads to genomes of *E. garzetta* [111] and *N*. *nycticorax*. There were about 60% to 75% of sequencing reads can be mapped to the reference genomes (Supplement Table S1 and S2). The mapping results of each sample transcripts were assembled by StringTie [112, 113], and then input to TACO [114] to reconstruct a consensus transcriptome by merging the biological repeat individual transcriptomes. The TACO results include the transcript of each gene and TPM of each group’s meta-assembly transcriptome merged from the biological repeats. This meta-assembly process can improve the precision of the transcript abundance [114]. OR genes expression levels were extracted from the TACO results according to functional annotation results.

## Supplementary Information

**Additional file 1:** **Supplement Fig 1.** K-mer distribution. The peak of 17-kmer located in Depth=45 and have 58,326,594,430 kmers. The raw genome size estimated by 17-kmer was 58,326,594,430/45=~1296.15Mb, and the revised genome size was 1272.61Mb by raw genome size* (1-E), where E represent the error rate estimate by Kdepth=1 rate. **Supplement Fig 2.** Expression numbers of ORs. Numbers of Expressed ORs in *E. garzetta*, and *N. nycticorax*. The numbers above the bars represent the expression numbers of ORs. **Supplement Table 1.** Transcriptome reads mapping in *N. nycticorax*. **Supplement Table 2.** Transcriptome reads mapping in *E. garzetta*. **Supplement Table 3.** Genome assembly statistics details. **Supplement Table 4.** Function of the visual adaptive evolution genes. **Supplement Table 5.** Positively selected sites in intact OR14 of the *E*. *garzetta*. **Supplement Table 6.** Positively selected sites in intact OR14 of the *N. nycticorax*. **Supplement Table 7.** Positively selected sites in intact OR14 of the *C. cochlearius*.

**Additional file 2:** **Supplement Table S1. **xlsx

**Additional file 3:** **Supplement Table S2. **xlsx

**Additional file 4: Supplement Table S3. **xlsx

## Data Availability

The draft genome assembly data are available at GenBank with the accession number: JAKFQP000000000. The associated BioProject number are PRJNA796267. RNA-Seq data are available at GEO with the accession number: GSE197206. The genomic annotation flies for genomic and transcriptomic analyses are available at https://github.com/hrluo93/4Herons-genome-anno.git
