# Supplementary material for: Genomic insight into the nocturnal adaptation of the black-crowned night heron (Nycticorax nycticorax)
Source: BMC Genomics. 2022 Oct 3;23:683. doi: 10.1186/s12864-022-08904-y (PMC9531477; doi:10.1186/s12864-022-08904-y)
Supplement: Supplementary file 1 — Additional file 1: Supplement Fig 1. K-mer distribution. The peak of 17-kmer located in Depth=45 and have 58,326,594,430 kmers. The raw genome size estimated by 17-kmer was 58,326,594,430/45=~1296.15Mb, and the revised genome size was 1272.61Mb by raw genome size* (1-E), where E represent the error rate estimate by Kdepth=1 rate. Supplement Fig 2. Expression numbers of ORs. Numbers of Expressed ORs in E. garzetta, and N. nycticorax. The numbers above the bars represent the expression numbers of ORs. Supplement Table 1. Transcriptome reads mapping in N. nycticorax. Supplement Table 2. Transcriptome reads mapping in E. garzetta. Supplement Table 3. Genome assembly statistics details. Supplement Table 4. Function of the visual adaptive evolution genes. Supplement Table 5. Positively selected sites in intact OR14 of the E. garzetta. Supplement Table 6. Positively selected sites in intact OR14 of the N. nycticorax. Supplement Table 7. Positively selected sites in intact OR14 of the C. cochlearius. [file 12864_2022_8904_MOESM1_ESM.docx]

**Genomic insight into nocturnal adaptation of the black-crowned night heron (*Nycticorax nycticorax*)**

**Haoran Luo, Site Luo, Wenzhen Fang, Qingxian Lin, Xiaolin Chen*, Xiaoping Zhou***

^1^Key Laboratory of Ministry of Education for Coast and Wetland Ecosystems, College of the Environment and Ecology, Xiamen University, Xiamen 361102, People’s Republic of China

*** Correspondence:**Xiaolin Chen

xlchen@xmu.edu.cn

Xiaoping Zhou

xpzhou@xmu.edu.cn

**
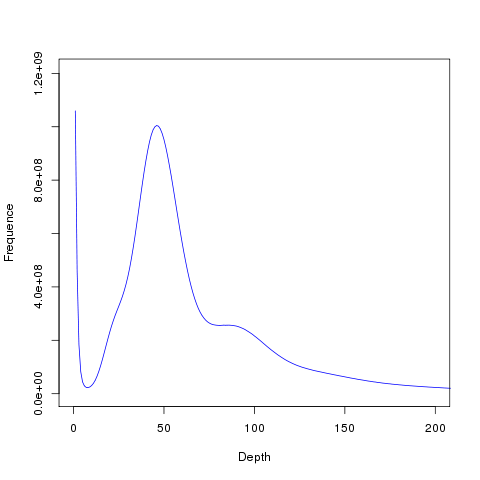
**

**Supplement Fig 1. K-mer distribution.** The peak of 17-kmer located in Depth=45 and have 58,326,594,430 kmers. The raw genome size estimated by 17-kmer was 58,326,594,430/45=~1296.15Mb, and the revised genome size was 1272.61Mb by raw genome size* (1-E), where E represent the error rate estimate by Kdepth=1 rate.

**
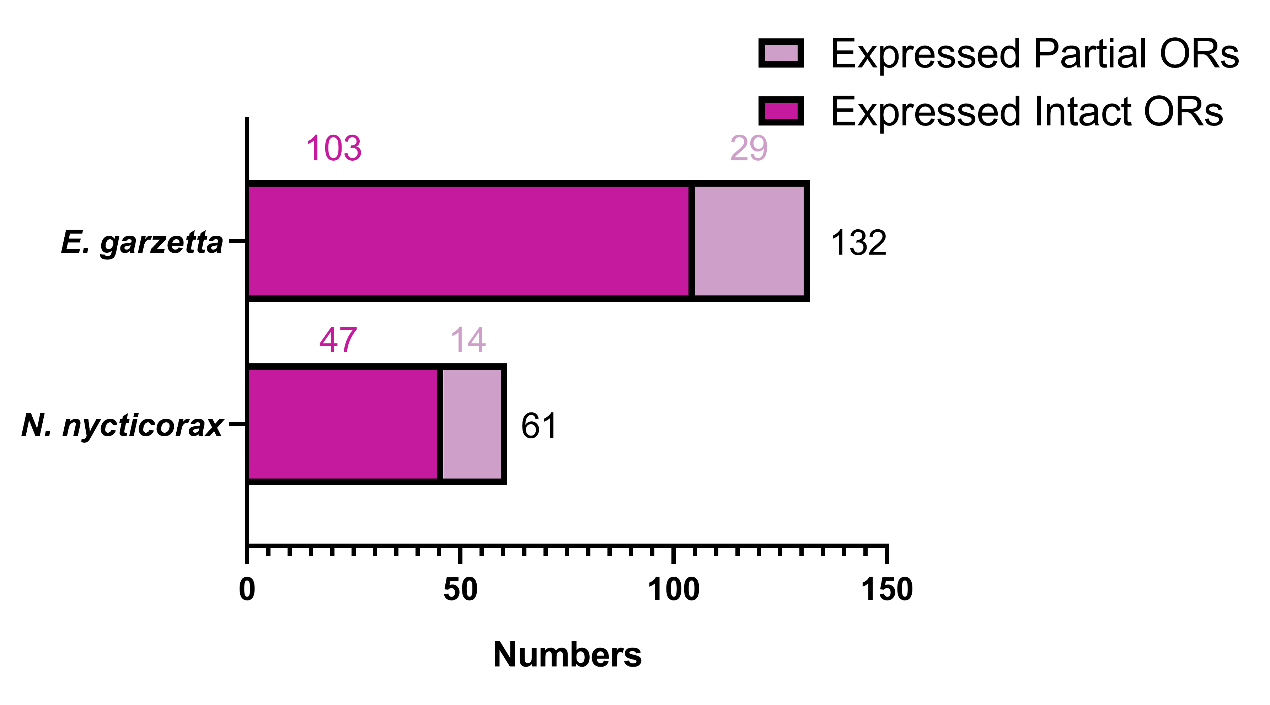
**

**Supplement Fig 2. Expression numbers of ORs.** Numbers of Expressed ORs in *E. garzetta*, and *N. nycticorax*. The numbers above the bars represent the expression numbers of ORs.

**Supplement Table 1.** Transcriptome reads mapping in *N. nycticorax*.

| Sample | Total reads | Total mapped | Uniquely mapped% |
| --- | --- | --- | --- |
| NN2OE  NN3OE  NN1OE  NN4OE  NN5OE  NN6OE | 31649915  26868635  22107587  26142198  27161451  23707660 | 57.59%  67.47%  70.21%  65.16%  63.03%  67.24% | 16128812 (50.96%)  16207270 (60.32%)  13946795 (63.09%)  14964649 (57.24%)  15105865 (55.62%)  14197635 (59.89%) |

**Supplement Table 2.** Transcriptome reads mapping in *E. garzetta*.

| Sample | Total paired reads | Total mapped | | Uniquely mapped |
| --- | --- | --- | --- | --- |
| EG1OE  EG2OE  EG3OE  EG4OE  EG5OE  EG6OE | 27202746  22506226  31591152  25221822  28409521  25374108 | 64.45%  74.37% 74.45%  73.07%  65.66%  69.33% | 14353084 (52.76%)  14477441 (64.33%)  20223040 (64.01%)  15773325 (62.54%)  15113041 (53.20%)  15066977 (59.38%) | |

**Supplement Table 3. Genome assembly statistics details**

| Index | Contig(bp) | Scaffold(bp) | Contig Number | | Scaffold Number |
| --- | --- | --- | --- | --- | --- |
| Total  Max  Length>=100  Length>=2000  N50  N60  N70  N80  N90 | 1,116,931,249 850,257  58,550  45,336  33,689  23,204  12,579 | 1,179,048,687 17,752,090  3,016,563  2,330,832  1,539,151  884,092  347,590 | | 46,539  46,539  38,451  5,277  7,449  10,309  14,286  20,711 | 7,611  7,611  4,549  104  148  211  312  518 |

**Supplement Table 4. Function of the visual adaptive evolution genes**

| Gene | Eye part | Disease | Function report and phenotype mutation consequence |
| --- | --- | --- | --- |
| *ADAMTS18*  *ALDH1A3*  *ATP8A2*  *CACNB2*  *CCDC66*  *CDON*  *CHD7*  *CRB1*  *CRYBA1*  *CTNS*  *EPAS1*  *EPHB1*  *FAT3*  *FGFR2*  *FOXP2*  *GNA11*  *GNAQ*  *GNAT1*  *HDAC1*  *HPS1*  *IMPG1 IMPG2*  *JAG1*  *MAP3K1*  *MDM1*  *MED1*  *MEGF11*  *MFN2*  *MITF*  *MYO7A*  *NHS*  *OLFM3*  *OPA1*  *OPN4*  *PAX6*  *PDE6B*  *PDGFRB*  *RNF2*  *RPE65*  *RPL24*  *RRM1*  *SH3PXD2B*  *SKI*  *SLC25A25*  *SLC4A10*  *SLC7A11*  *SMAD3*  *SOX14*  *TTC8*  *TULP3*  *UCHL3*  *UNC119*  *VEGFA*  *WDR19* | Cornea and Retina  Whole eye-small size eye microphthalmia  Retina  -  Retina  Whole eye-small size eye microphthalmia  Retina  Retina  Lens  Corneal  Retina  Retina  Retina  Whole-Bulging eyes  Eyelids  Intraocular changes-choroid, ciliary body, iris  Retina  Retina  Intraocular changes-choroid, ciliary body, iris  Retina  Lens  Retina  Retina  Lens and retina  Retina  Lens and cornea  Iris and Retina  Retina  Lens  Intraocular pressure  Optic nerve  Retina  Iris and Optic nerve  Retina  Retina  Whole eye-small size eye microphthalmia  Retina  Retina  Retina  Cornea  Whole eye-Protruding eyes  Retina  Corneal  Retina  Lens  Eyelids  Retina  Pressure inside retina  Retina  Retina  Choroid and retina  Retina | Microcornea, Myopic Chorioretinal Atrophy, and Telecanthus  Microphthalmia  Cone-Rod Dystrophy  Blindness  Retinal Degeneration  Microphthalmia  CHARGE syndrome  Retinitis pigmentosa  Congenital zonular cataract with sutural opacities  Adult nonnephropathic cystinosis; late-onset juvenile or adolescent nephropathic type cystinosis; nephropathic cystinosis  Retinal Hemangioblastoma  Retinal ganglion cell axon guidance  Retina development  Apert syndrome; Crouzon syndrome  Blepharophimosis  Ocular Melanoma  Ocular Melanoma  Autosomal dominant congenital stationary night blindness  Retinoblastoma  Hermansky–Pudlak syndrome (oculocutaneous albinism)  Retinitis Pigmentosa  Lens progenitor cell deficits.  Retinal Degeneration  Retinal Degeneration  Retinoblastoma; Microphthalmia  Retinal neuronal arrays  Optic Atrophy; Intraocular Pressure Quantitative Trait Locus  Tietz syndrome; Waardenburg syndrome type II  Usher syndrome type 1B  Cataract-dental syndrome  Glaucoma, Primary Open Angle  Optic atrophy-1  Achromatopsia  Aniridia type II; Coloboma of optic nerve; Opic nerve hypoplasia  Congenital stationary night blindness  Retinitis Pigmentosa  Microphthalmia  Congenital stationary night blindness  Retinitis Pigmentosa  Retinitis Pigmentosa  Frank-Ter Haar Syndrome  Shprintzen-Goldberg Craniosynostosis Syndrome  Fundus Dystrophy  Corneal Dystrophy  Oxidant-induced cellular damage in retina  Cataract  Colobomatous Microphthalmia  Bardet-Biedl syndrome  Glaucoma  Retinal Degeneration  Dominant cone-rod dystrophy  Retinal Vascular Disease  Autosomal recessive retinitis pigmentosa | ADAMTS18 plays an essential role in early eye development and those mutations therein cause a distinct eye phenotype that is mainly characterized by microcornea and myopia[1]. Also related retinal detachment.  Loss of function of ALDH1A3 loss of function causes bilateral anophthalmia/microphthalmia caused bilateral anophthalmia/microphthalmia[2, 3]  ATP8A2-deficient mice have shortened photoreceptor outer segments, cause a reduction in light responses and decreased photoreceptor viability[4]  Mutations in *CACNB2* cause incomplete congenital stationary night blindness. Under dark-adapted conditions, CNS-beta (2)-null mice did not have a normal b-wave[5]  Related to the retinal rod cell development. Protein CCDC66 is expressed predominantly in the developing rod outer segments related retinal degeneration and dysfunction and detection of light stimulus involved in visual perception[6]  CDON related to embryonic retina morphogenesis, and mice lacking CDON display multiple eye defects[7]  *CHD7* regulates the differentiation of retinal cells and plays an essential role in retinal cell development. Mutations in *CHD7* cause abnormal expression or a complete absence of molecular markers for the retinal ganglion cells and photoreceptors[8]  Mutations in the *CRB1* gene result in human retinal diseases including retinitis pigmentosa and Leber congenital amaurosis[9, 10]  *CRYBA1* encode the Beta-crystallin A1, crystallin are major protein constituents of the mammalian lens, where Beta-crystallin stability association into higher order complexes are critical for lens clarity and refraction[11]  The cystinosin (Ctns(-/-)) mouse related the cystinosis. Mouse begin to die in 7-12 months because of the cystinosis disease increased Corneal crystals and corneas become scarred and neovascularized[12]  Mutations in mice related to exhibited marked thinning of the retina and abnormal retinal vasculature[13]  EPHB1 is responsible for the retinal axon guidance, redirecting the retinal ganglion cells axons at the optic chiasm midline[14]  FAT3 ensures that retinal amacrine cells project develop primary dendrites into the inner plexiform layer (IPL) and only rarely extend processes into other retinal layers[15]  FGF receptors are essential for lens fiber differentiation, different FGF receptors function redundantly[16]  Mutations in FOXP2 related abnormal narrowness of the palpebral fissure in the horizontal direction caused by the lateral displacement of the medial canthi of the eyelids[17]  Related intraocular melanoma which is a cancer of the pigment-producing cells (melanocytes) in the middle layer of the eye, usually in choroid, ciliary body, or iris[18], associated with GNAQ[19]  Related to Sturge-Weber syndrome[20] and ocular melanoma[18, 19]  GNAT1 Stimulates the coupling of rhodopsin and cGMP-phoshodiesterase during visual impulses. Mutations in this gene result in autosomal dominant congenital stationary night blindness[21]  Related to retinoblastoma (RB1), caused blindness or poor vision in the affected eye or eyes[22]  This gene encodes a protein that may play a role in organelle biogenesis associated with melanosomes, platelet dense granules, and lysosomes and mutations in HSP1 caused pigmentation defect in the eyes[23]  IMPG1 and IMPG2 are members of the interphotoreceptor matrix, which is occupying the interface between photoreceptors and the retinal pigment epithelium in the fundus of the eye. The IMPG matrix is a unique extracellular complex that play a supportive role in photoreceptor maintenance[24]  JAG1 mutants have both lens progenitor cell proliferation and differentiation deficits[25, 26]  MAP3K1 played an essential role during mouse embryonic eyelid closure, Homozygous deletion of either gene causes defective eyelid closure[27, 28] and progressive and eventual death of the cells of the retina[29]  *Mdm1* mutations related the age-related retinal degeneration[30]  MED1 is critical for the lens development in camera-type eye and related in paucity of retinal pigment, defective lens formation[31]  MEGF11 played critical roles in the formation of mosaics in the retina[32]  *MFN2* caused small, opacified lens and small eyeball in the most severe phenotypes[33] and deletion affects corneal development because of collagen hyperplasia in the corneal stroma[34]  Involved in melanin synthesis. Related to Waardenburg syndrome type 2 (WS2), mutations caused ocular albinism[35, 36]  MYO7A present in the retinal pigment epithelium and plays an important role in regulating opsin transport in retinal photoreceptors.[37, 38]  Mutation causing X-linked dominant congenital cataract[39]  Olfactomedin 3 might be required for normal development and function of tissues involved in glaucoma, eye angle and retina. Mutations in the *OLFM3* may lead to different eye pathologies[40]  Related to optic atrophy type 1, a disease that affects the optic nerve. Caused vision loss, difficulty distinguishing colors, and an abnormally pale appearance (pallor) of the optic nerve[41, 42]  Mutations related to retinal degeneration because of dystrophy[43, 44]  Related to aniridia, an eye disorder characterized by a complete or partial absence of the colored part of the iris[45]. PAX6 gene also associated with a variety of optic-nerve malformations[46]  PDE6B related to retinitis pigmentosa and cause progressive vision loss[47]  Related to retina vasculature development in camera-type eye[48]  During eye development, Bcl6a with Rnf2 prevent eye colobomata (Microphthalmia)[49]  The RPE65 protein plays a role in vitamin A metabolism in the retina, and the whole region of the protein is highly conserved in vertebrates[50], mutations in the RPE65 cause Leber’s congenital amaurosis[51, 52] and early-onset retinal degeneration[53]  The Belly spot and tail (Bst) is a semidominant mouse mutation that disrupts the retinal cell fate determination and as a deletion within the *RPL24* gene[54]. Mutant phenotype of Bst affected retinas are smaller than wildtype[55]  Related to retinal pigment deposits and photoreceptor cells loss[56]  Encodes the TKS4 protein. Mice lacking Tks4 showed pronounced skeletal, eye, and cardiac abnormalities and phenocopied most of the defects associated with Frank-Ter Haar syndrome. SH3PXD2B mutations caused prominent eyes, macrocornea with or without glaucoma[57, 58]  Cause prominent eyes due to Shprintzen-Goldberg Craniosynostosis Syndrome[59]  Related to retinal dystrophy[56]  Related to corneal dystrophy[56]  SLC7A11 (system xc −) is a transport system critical to potentiation of antioxidant signaling in retina[60]  Related to the coordination of cell cycle progression and the initiation of lens differentiation[61]  Related to abnormal narrowness of the palpebral fissure in the horizontal direction caused by the lateral displacement of the medial canthi of the eyelids[56]  Related to Bardet-Biedl Syndrome. One of the major features of Bardet-Biedl syndrome is vision loss. Occurs as the light-sensing tissue at the back of the eye (the retina) gradually deteriorates[62, 63]  Disruption of mouse TULP3 caused morphological defects in the embryonic craniofacial regions and eye defects (retina)[64]  Associated to retina development[23]  Related to Cone-rod dystrophyand causes vision loss[65, 66]  Related choroid development in eye and inactivation of *VEGF* expression results in the absence of choriocapillaris, occurrence of microphthalmia, and the loss of visual function[67]  WDR19 related to photoreceptor intra-flagellar transport function and was shown to accumulate along the connecting cilia in Nphp1 photoreceptors[68] |

**Supplement Table 5. Positively selected sites in intact OR14 of the *E. garzetta***

| MEME (Q-value< 0.05) | FUBAR (BayesFactor> 0.95) | SLAC (P [dN/dS > 1] < 0.1) | |
| --- | --- | --- | --- |
| 8, 14, 20, 28, 52, 78, 93, 106, 107, 110, 145, 153, 155, 156, 196, 199, 204, 205, 206, 211, 215, 230, 251, 254, 260, 282, 283 | 52, 93, 107, 110, 196, 199, 204, 211, 251, 283 | 1, 3, 5, 8, 10, 11, 12, 14, 15, 17, 25, 26, 39, 40, 44, 45, 46, 49, 52, 55, 56, 57, 59, 61, 63, 64, 67, 68, 70, 73, 74, 77, 79, 83, 84, 88, 90, 91, 92, 94, 95, 97, 98, 99, 104, 107, 109, 114, 115, 118, 119, 121, 128, 130, 132, 133, 150, 151, 155, 157, 158, 160, 163, 164, 165, 167, 168, 170, 171, 174, 176, 177, 178  179, 180, 185, 187, 189, 192, 195, 196, 198, 200, 201, 204, 208, 210, 213, 215, 216, 217, 220, 221, 223, 224, 230, 232, 237, 239, 240, 241, 242, 243, 244, 245, 246, 247, 248, 249, 250, 251, 253, 259, 261, 262, 263, 265, 266, 269, 270, 271, 273, 274, 275, 276, 277, 278, 279, 280, 281, 282, 283, 285, 286, 288, 289, 290, 293, 295, 296, 297, 299, 300, 302 |  |

Site numbers with underline represent this site detected positive selection by all methods.

**Supplement Table 6. Positively selected sites in intact OR14 of the *N. nycticorax***

| MEME (Q-value< 0.05) | FUBAR (BayesFactor> 0.95) | SLAC (P [dN/dS > 1] < 0.1) | |
| --- | --- | --- | --- |
| 93, 110 | 52, 93, 107, 110, 211, 284 | 3, 4, 8, 10, 14, 15, 17, 18, 36, 39, 40, 49, 52, 59, 67, 70, 73, 77, 83, 84, 88, 89, 90, 91, 92, 93, 94, 95, 97, 98, 104, 107, 109, 110, 114, 118, 121, 125, 130, 133, 150, 151, 158, 161, 163, 164, 165, 168, 171, 180, 189, 195, 200, 201, 2 |  |

Site numbers with underline represent this site detected positive selection by all methods.

**Supplement Table 7. Positively selected sites in intact OR14 of the *C. cochlearius***

| MEME (Q-value< 0.05) | FUBAR (BayesFactor> 0.95) | SLAC (P [dN/dS > 1] < 0.1) | |
| --- | --- | --- | --- |
| 6, 14, 16, 24, 26, 28, 29, 30, 33, 35, 47, 68, 83, 93, 101, 107, 110, 128, 150, 154, 155, 160, 172, 196, 202, 204, 205, 206, 207, 215, 267, 283, 284, 294, 301 | 16, 47, 93, 107, 153, 172, 196, 204, 207, 283 | 3, 4, 8, 12, 14, 15, 16, 17, 18, 23, 36, 38, 39, 40, 45, 46, 47, 49, 52, 61, 65, 66, 67, 70, 73, 75, 77, 84, 87, 88, 90, 91, 92, 94, 95, 97, 98, 100, 104, 107, 109, 112, 113, 114, 115, 118, 121, 127, 130, 132, 139, 150, 151, 155, 158, 160, 162, 163, 164, 165, 168, 171, 172, 180, 187, 189, 192, 193, 194, 195, 196, 197, 201, 202, 204, 207, 208, 213, 216, 217, 223, 229, 232, 239, 241, 245, 248, 249, 252, 253, 258, 259, 260, 261, 262, 263, 265, 268, 269, 270, 271, 272, 274, 275, 277, 278, 279, 280, 281, 282, 283  284, 286, 289, 290, 293, 295, 297, 299, 300, 302 |  |

Site numbers with underline represent this site detected positive selection by all methods.

1. Aldahmesh MA, Alshammari MJ, Khan AO, Mohamed JY, Alhabib FA, Alkuraya FS: **The syndrome of microcornea, myopic chorioretinal atrophy, and telecanthus (MMCAT) is caused by mutations in ADAMTS18**. *Hum Mutat* 2013, **34**(9):1195-1199.

2. Fares-Taie L, Gerber S, Chassaing N, Clayton-Smith J, Hanein S, Silva E, Serey M, Serre V, Gerard X, Baumann C *et al*: **ALDH1A3 mutations cause recessive anophthalmia and microphthalmia**. *Am J Hum Genet* 2013, **92**(2):265-270.

3. Yahyavi M, Abouzeid H, Gawdat G, de Preux AS, Xiao T, Bardakjian T, Schneider A, Choi A, Jorgenson E, Baier H *et al*: **ALDH1A3 loss of function causes bilateral anophthalmia/microphthalmia and hypoplasia of the optic nerve and optic chiasm**. *Hum Mol Genet* 2013, **22**(16):3250-3258.

4. Coleman JA, Zhu X, Djajadi HR, Molday LL, Smith RS, Libby RT, John SW, Molday RS: **Phospholipid flippase ATP8A2 is required for normal visual and auditory function and photoreceptor and spiral ganglion cell survival**. *J Cell Sci* 2014, **127**(Pt 5):1138-1149.

5. Ball SL, Powers PA, Shin HS, Morgans CW, Peachey NS, Gregg RG: **Role of the beta(2) subunit of voltage-dependent calcium channels in the retinal outer plexiform layer**. *Invest Ophthalmol Vis Sci* 2002, **43**(5):1595-1603.

6. Gerding WM, Schreiber S, Schulte-Middelmann T, de Castro Marques A, Atorf J, Akkad DA, Dekomien G, Kremers J, Dermietzel R, Gal A *et al*: **Ccdc66 null mutation causes retinal degeneration and dysfunction**. *Hum Mol Genet* 2011, **20**(18):3620-3631.

7. Zhang W, Mulieri PJ, Gaio U, Bae GU, Krauss RS, Kang JS: **Ocular abnormalities in mice lacking the immunoglobulin superfamily member Cdo**. *FEBS J* 2009, **276**(20):5998-6010.

8. Patten SA, Jacobs-McDaniels NL, Zaouter C, Drapeau P, Albertson RC, Moldovan F: **Role of Chd7 in zebrafish: a model for CHARGE syndrome**. *PLoS One* 2012, **7**(2):e31650.

9. den Hollander AI, ten Brink JB, de Kok YJ, van Soest S, van den Born LI, van Driel MA, van de Pol DJ, Payne AM, Bhattacharya SS, Kellner U *et al*: **Mutations in a human homologue of Drosophila crumbs cause retinitis pigmentosa (RP12)**. *Nat Genet* 1999, **23**(2):217-221.

10. den Hollander AI, Heckenlively JR, van den Born LI, de Kok YJ, van der Velde-Visser SD, Kellner U, Jurklies B, van Schooneveld MJ, Blankenagel A, Rohrschneider K *et al*: **Leber congenital amaurosis and retinitis pigmentosa with Coats-like exudative vasculopathy are associated with mutations in the crumbs homologue 1 (CRB1) gene**. *Am J Hum Genet* 2001, **69**(1):198-203.

11. Sergeev YV, Hejtmancik JF, Wingfield PT: **Energetics of domain-domain interactions and entropy driven association of beta-crystallins**. *Biochemistry* 2004, **43**(2):415-424.

12. Simpson J, Nien CJ, Flynn K, Jester B, Cherqui S, Jester J: **Quantitative in vivo and ex vivo confocal microscopy analysis of corneal cystine crystals in the Ctns knockout mouse**. *Mol Vis* 2011, **17**:2212-2220.

13. Ding K, Scortegagna M, Seaman R, Birch DG, Garcia JA: **Retinal disease in mice lacking hypoxia-inducible transcription factor-2alpha**. *Invest Ophthalmol Vis Sci* 2005, **46**(3):1010-1016.

14. Chenaux G, Henkemeyer M: **Forward signaling by EphB1/EphB2 interacting with ephrin-B ligands at the optic chiasm is required to form the ipsilateral projection**. *Eur J Neurosci* 2011, **34**(10):1620-1633.

15. Deans MR, Krol A, Abraira VE, Copley CO, Tucker AF, Goodrich LV: **Control of neuronal morphology by the atypical cadherin Fat3**. *Neuron* 2011, **71**(5):820-832.

16. Zhao H, Yang T, Madakashira BP, Thiels CA, Bechtle CA, Garcia CM, Zhang H, Yu K, Ornitz DM, Beebe DC *et al*: **Fibroblast growth factor receptor signaling is essential for lens fiber cell differentiation**. *Dev Biol* 2008, **318**(2):276-288.

17. Prueitt RL, Zinn AR: **A fork in the road to fertility**. *Nat Genet* 2001, **27**(2):132-134.

18. MacConaill LE, Garcia E, Shivdasani P, Ducar M, Adusumilli R, Breneiser M, Byrne M, Chung L, Conneely J, Crosby L *et al*: **Prospective enterprise-level molecular genotyping of a cohort of cancer patients**. *J Mol Diagn* 2014, **16**(6):660-672.

19. Chen X, Wu Q, Tan L, Porter D, Jager MJ, Emery C, Bastian BC: **Combined PKC and MEK inhibition in uveal melanoma with GNAQ and GNA11 mutations**. *Oncogene* 2014, **33**(39):4724-4734.

20. Shirley MD, Tang H, Gallione CJ, Baugher JD, Frelin LP, Cohen B, North PE, Marchuk DA, Comi AM, Pevsner J: **Sturge-Weber syndrome and port-wine stains caused by somatic mutation in GNAQ**. *N Engl J Med* 2013, **368**(21):1971-1979.

21. Naeem MA, Chavali VR, Ali S, Iqbal M, Riazuddin S, Khan SN, Husnain T, Sieving PA, Ayyagari R, Riazuddin S *et al*: **GNAT1 associated with autosomal recessive congenital stationary night blindness**. *Invest Ophthalmol Vis Sci* 2012, **53**(3):1353-1361.

22. LeBoeuf M, Terrell A, Trivedi S, Sinha S, Epstein JA, Olson EN, Morrisey EE, Millar SE: **Hdac1 and Hdac2 act redundantly to control p63 and p53 functions in epidermal progenitor cells**. *Dev Cell* 2010, **19**(6):807-818.

23. Won J, Shi LY, Hicks W, Wang J, Hurd R, Naggert JK, Chang B, Nishina PM: **Mouse model resources for vision research**. *J Ophthalmol* 2011, **2011**:391384.

24. Acharya S, Foletta VC, Lee JW, Rayborn ME, Rodriguez IR, Young WS, 3rd, Hollyfield JG: **SPACRCAN, a novel human interphotoreceptor matrix hyaluronan-binding proteoglycan synthesized by photoreceptors and pinealocytes**. *J Biol Chem* 2000, **275**(10):6945-6955.

25. Oda T, Elkahloun AG, Pike BL, Okajima K, Krantz ID, Genin A, Piccoli DA, Meltzer PS, Spinner NB, Collins FS *et al*: **Mutations in the human Jagged1 gene are responsible for Alagille syndrome**. *Nat Genet* 1997, **16**(3):235-242.

26. Le TT, Conley KW, Brown NL: **Jagged 1 is necessary for normal mouse lens formation**. *Dev Biol* 2009, **328**(1):118-126.

27. Meng Q, Mongan M, Wang J, Tang X, Zhang J, Kao W, Xia Y: **Epithelial sheet movement requires the cooperation of c-Jun and MAP3K1**. *Dev Biol* 2014, **395**(1):29-37.

28. Jin C, Chen J, Meng Q, Carreira V, Tam NN, Geh E, Karyala S, Ho SM, Zhou X, Medvedovic M *et al*: **Deciphering gene expression program of MAP3K1 in mouse eyelid morphogenesis**. *Dev Biol* 2013, **374**(1):96-107.

29. Mongan M, Wang J, Liu H, Fan Y, Jin C, Kao WY, Xia Y: **Loss of MAP3K1 enhances proliferation and apoptosis during retinal development**. *Development* 2011, **138**(18):4001-4012.

30. Chang B, Mandal MN, Chavali VR, Hawes NL, Khan NW, Hurd RE, Smith RS, Davisson ML, Kopplin L, Klein BE *et al*: **Age-related retinal degeneration (arrd2) in a novel mouse model due to a nonsense mutation in the Mdm1 gene**. *Hum Mol Genet* 2008, **17**(24):3929-3941.

31. Crawford SE, Qi C, Misra P, Stellmach V, Rao MS, Engel JD, Zhu Y, Reddy JK: **Defects of the heart, eye, and megakaryocytes in peroxisome proliferator activator receptor-binding protein (PBP) null embryos implicate GATA family of transcription factors**. *J Biol Chem* 2002, **277**(5):3585-3592.

32. Kay JN, Chu MW, Sanes JR: **MEGF10 and MEGF11 mediate homotypic interactions required for mosaic spacing of retinal neurons**. *Nature* 2012, **483**(7390):465-469.

33. Zhang J, Qu B, Yu W, Zhu Y, Yan X, Shen H, Zhao J: **Role of surface ectoderm-specific mitofusin 2 in the corneal morphologic development of mice**. *Am J Transl Res* 2019, **11**(6):3620-3628.

34. Zhao J, Wu X, Wu D, Yu Y, Yu Y, Wang Y, Fu Q, Zhang J, Yao K: **Embryonic Surface Ectoderm-specific Mitofusin 2 Conditional Knockout Induces Congenital Cataracts in Mice**. *Sci Rep* 2018, **8**(1):1522.

35. Bard LA: **Heterogeneity in Waardenburg's syndrome. Report of a family with ocular albinism**. *Arch Ophthalmol* 1978, **96**(7):1193-1198.

36. Tassabehji M, Newton VE, Read AP: **Waardenburg syndrome type 2 caused by mutations in the human microphthalmia (MITF) gene**. *Nat Genet* 1994, **8**(3):251-255.

37. Hasson T, Heintzelman MB, Santos-Sacchi J, Corey DP, Mooseker MS: **Expression in cochlea and retina of myosin VIIa, the gene product defective in Usher syndrome type 1B**. *Proc Natl Acad Sci U S A* 1995, **92**(21):9815-9819.

38. Weston MD, Kelley PM, Overbeck LD, Wagenaar M, Orten DJ, Hasson T, Chen ZY, Corey D, Mooseker M, Sumegi J *et al*: **Myosin VIIA mutation screening in 189 Usher syndrome type 1 patients**. *Am J Hum Genet* 1996, **59**(5):1074-1083.

39. Grimes PA, Favor J, Koeberlein B, Silvers WK, Fitzgerald PG, Stambolian D: **Lens development in a dominant X-linked congenital cataract of the mouse**. *Exp Eye Res* 1993, **57**(5):587-594.

40. Torrado M, Trivedi R, Zinovieva R, Karavanova I, Tomarev SI: **Optimedin: a novel olfactomedin-related protein that interacts with myocilin**. *Hum Mol Genet* 2002, **11**(11):1291-1301.

41. Votruba M, Moore AT, Bhattacharya SS: **Demonstration of a founder effect and fine mapping of dominant optic atrophy locus on 3q28-qter by linkage disequilibrium method: a study of 38 British Isles pedigrees**. *Hum Genet* 1998, **102**(1):79-86.

42. Johnston RL, Seller MJ, Behnam JT, Burdon MA, Spalton DJ: **Dominant optic atrophy**. *Ophthalmology* 1999, **106**(1):123-128.

43. Vugler AA, Semo M, Joseph A, Jeffery G: **Survival and remodeling of melanopsin cells during retinal dystrophy**. *Vis Neurosci* 2008, **25**(2):125-138.

44. Lin B, Koizumi A, Tanaka N, Panda S, Masland RH: **Restoration of visual function in retinal degeneration mice by ectopic expression of melanopsin**. *Proc Natl Acad Sci U S A* 2008, **105**(41):16009-16014.

45. Jordan T, Hanson I, Zaletayev D, Hodgson S, Prosser J, Seawright A, Hastie N, van Heyningen V: **The human PAX6 gene is mutated in two patients with aniridia**. *Nat Genet* 1992, **1**(5):328-332.

46. Azuma N, Yamaguchi Y, Handa H, Tadokoro K, Asaka A, Kawase E, Yamada M: **Mutations of the PAX6 gene detected in patients with a variety of optic-nerve malformations**. *Am J Hum Genet* 2003, **72**(6):1565-1570.

47. McLaughlin ME, Sandberg MA, Berson EL, Dryja TP: **Recessive mutations in the gene encoding the beta-subunit of rod phosphodiesterase in patients with retinitis pigmentosa**. *Nat Genet* 1993, **4**(2):130-134.

48. Tallquist MD, French WJ, Soriano P: **Additive effects of PDGF receptor beta signaling pathways in vascular smooth muscle cell development**. *PLoS Biol* 2003, **1**(2):E52.

49. Lee J, Lee BK, Gross JM: **Bcl6a function is required during optic cup formation to prevent p53-dependent apoptosis and colobomata**. *Hum Mol Genet* 2013, **22**(17):3568-3582.

50. Nicoletti A, Wong DJ, Kawase K, Gibson LH, Yang-Feng TL, Richards JE, Thompson DA: **Molecular characterization of the human gene encoding an abundant 61 kDa protein specific to the retinal pigment epithelium**. *Hum Mol Genet* 1995, **4**(4):641-649.

51. Morimura H, Fishman GA, Grover SA, Fulton AB, Berson EL, Dryja TP: **Mutations in the RPE65 gene in patients with autosomal recessive retinitis pigmentosa or leber congenital amaurosis**. *Proc Natl Acad Sci U S A* 1998, **95**(6):3088-3093.

52. Kondo H, Qin M, Mizota A, Kondo M, Hayashi H, Hayashi K, Oshima K, Tahira T, Hayashi K: **A homozygosity-based search for mutations in patients with autosomal recessive retinitis pigmentosa, using microsatellite markers**. *Invest Ophthalmol Vis Sci* 2004, **45**(12):4433-4439.

53. Gu SM, Thompson DA, Srikumari CR, Lorenz B, Finckh U, Nicoletti A, Murthy KR, Rathmann M, Kumaramanickavel G, Denton MJ *et al*: **Mutations in RPE65 cause autosomal recessive childhood-onset severe retinal dystrophy**. *Nat Genet* 1997, **17**(2):194-197.

54. Oliver ER, Saunders TL, Tarle SA, Glaser T: **Ribosomal protein L24 defect in belly spot and tail (Bst), a mouse Minute**. *Development* 2004, **131**(16):3907-3920.

55. Tang Q, Rice DS, Goldowitz D: **Disrupted retinal development in the embryonic belly spot and tail mutant mouse**. *Dev Biol* 1999, **207**(1):239-255.

56. Rappaport N, Twik M, Plaschkes I, Nudel R, Iny Stein T, Levitt J, Gershoni M, Morrey CP, Safran M, Lancet D: **MalaCards: an amalgamated human disease compendium with diverse clinical and genetic annotation and structured search**. *Nucleic Acids Res* 2017, **45**(D1):D877-D887.

57. Maas SM, Kayserili H, Lam J, Apak MY, Hennekam RC: **Further delineation of Frank-ter Haar syndrome**. *Am J Med Genet A* 2004, **131**(2):127-133.

58. Iqbal Z, Cejudo-Martin P, de Brouwer A, van der Zwaag B, Ruiz-Lozano P, Scimia MC, Lindsey JD, Weinreb R, Albrecht B, Megarbane A *et al*: **Disruption of the podosome adaptor protein TKS4 (SH3PXD2B) causes the skeletal dysplasia, eye, and cardiac abnormalities of Frank-Ter Haar Syndrome**. *Am J Hum Genet* 2010, **86**(2):254-261.

59. Doyle AJ, Doyle JJ, Bessling SL, Maragh S, Lindsay ME, Schepers D, Gillis E, Mortier G, Homfray T, Sauls K *et al*: **Mutations in the TGF-beta repressor SKI cause Shprintzen-Goldberg syndrome with aortic aneurysm**. *Nat Genet* 2012, **44**(11):1249-1254.

60. Ananth S, Babu E, Veeranan-Karmegam R, Bozard Baldowski BR, Boettger T, Martin PM: **Induction of the cystine/glutamate exchanger SLC7A11 in retinal pigment epithelial cells by the antipsoriatic drug monomethylfumarate**. *Invest Ophthalmol Vis Sci* 2013, **54**(3):1592-1602.

61. Wu G, Glickstein S, Liu W, Fujita T, Li W, Yang Q, Duvoisin R, Wan Y: **The anaphase-promoting complex coordinates initiation of lens differentiation**. *Mol Biol Cell* 2007, **18**(3):1018-1029.

62. Stoetzel C, Laurier V, Faivre L, Megarbane A, Perrin-Schmitt F, Verloes A, Bonneau D, Mandel JL, Cossee M, Dollfus H: **BBS8 is rarely mutated in a cohort of 128 Bardet-Biedl syndrome families**. *J Hum Genet* 2006, **51**(1):81-84.

63. Smaoui N, Chaabouni M, Sergeev YV, Kallel H, Li S, Mahfoudh N, Maazoul F, Kammoun H, Gandoura N, Bouaziz A *et al*: **Screening of the eight BBS genes in Tunisian families: no evidence of triallelism**. *Invest Ophthalmol Vis Sci* 2006, **47**(8):3487-3495.

64. Norman RX, Ko HW, Huang V, Eun CM, Abler LL, Zhang Z, Sun X, Eggenschwiler JT: **Tubby-like protein 3 (TULP3) regulates patterning in the mouse embryo through inhibition of Hedgehog signaling**. *Hum Mol Genet* 2009, **18**(10):1740-1754.

65. Huang L, Xiao X, Li S, Jia X, Wang P, Sun W, Xu Y, Xin W, Guo X, Zhang Q: **Molecular genetics of cone-rod dystrophy in Chinese patients: New data from 61 probands and mutation overview of 163 probands**. *Exp Eye Res* 2016, **146**:252-258.

66. Kobayashi A, Higashide T, Hamasaki D, Kubota S, Sakuma H, An W, Fujimaki T, McLaren MJ, Weleber RG, Inana G: **HRG4 (UNC119) mutation found in cone-rod dystrophy causes retinal degeneration in a transgenic model**. *Invest Ophthalmol Vis Sci* 2000, **41**(11):3268-3277.

67. Marneros AG, Fan J, Yokoyama Y, Gerber HP, Ferrara N, Crouch RK, Olsen BR: **Vascular endothelial growth factor expression in the retinal pigment epithelium is essential for choriocapillaris development and visual function**. *Am J Pathol* 2005, **167**(5):1451-1459.

68. Jiang ST, Chiou YY, Wang E, Chien YL, Ho HH, Tsai FJ, Lin CY, Tsai SP, Li H: **Essential role of nephrocystin in photoreceptor intraflagellar transport in mouse**. *Hum Mol Genet* 2009, **18**(9):1566-1577.
